# Prognostic Impact of Residual Moderate Mitral Regurgitation Following Valve-in-Valve Transcatheter Aortic Valve Implantation

**DOI:** 10.21470/1678-9741-2023-0012

**Published:** 2023-10-23

**Authors:** Tomasz Stankowski, Sleiman Sebastian Aboul-Hassan, Mohammed Salem, Kristin Rochor, Soeren Schenk, Temirlan Erkenov, Farzaneh Seifi Zinab, Anja Muehle, Volker Herwig, Axel Harnath, Michel Pompeu Sá, Basel Ramlawi, Dirk Fritzsche, Bartłomiej Perek

**Affiliations:** 1 Department of Cardiac Surgery, Sana Heart Center Cottbus, Cottbus, Germany; 2 Department of Cardiac Surgery, Medinet Heart Center Ltd., Nowa Sol, Poland; 3 Department of Cardiac Surgery and Interventional Cardiology, Faculty of Medicine and Medical Sciences, University of Zielona Gora, Zielona Gora, Poland; 4 Department of Cardiac Surgery Research, Lankenau Institute for Medical Research, Main Line Health, Wynnewood, Pennsylvania, United States of America; 5 Department of Cardiothoracic Surgery, Lankenau Heart Institute, Main Line Health, Wynnewood, Pennsylvania, United States of America; 6 Department of Cardiac Surgery and Transplantology, Poznan University of Medical Sciences, Poznan, Poland

**Keywords:** Bioprosthesis, Mitral Valve Insufficiency, Mitral Valve, Retrospective Studies, Propensity Score, Prognosis, Follow-Up Studies, Survivors

## Abstract

**Introduction:**

The impact of mitral regurgitation (MR) on valve-in-valve transcatheter aortic valve implantation (VIV-TAVI) in patients with failed bioprostheses remains unclear. The purpose of this study was to assess the prognostic impact of residual moderate MR following VIV-TAVI.

**Methods:**

We retrospectively analyzed 127 patients who underwent VIV-TAVI between March 2010 and November 2021. At least moderate MR was observed in 51.2% of patients before the procedure, and MR improved in 42.1% of all patients. Patients with postoperative severe MR, previous mitral valve intervention, and patients who died before postoperative echocardiography were excluded from further analyses. The remaining 114 subjects were divided into two groups according to the degree of postprocedural MR: none-mild MR (73.7%) or moderate MR (26.3%). Propensity score matching yielded 23 pairs for final comparison.

**Results:**

No significant differences were found between groups before and after matching in early results. In the matched cohort, survival probabilities at one, three, and five years were 95.7% vs. 87.0%, 85.0% vs. 64.5%, and 85.0% vs. 29.0% in the none-mild MR group vs. moderate MR-group, respectively (log-rank P=0.035). Among survivors, patients with moderate MR had worse functional status according to New York Heart Association (NYHA) class at follow-up (P=0.006).

**Conclusion:**

MR is common in patients with failed aortic bioprostheses, and improvement in MR-status was observed in over 40% of patients following VIV-TAVI. Residual moderate MR after VIV-TAVI is not associated with worse early outcomes, however, it was associated with increased mortality at five years of follow-up and worse NYHA class among survivors.

## INTRODUCTION

Aortic stenosis and mitral regurgitation (MR) are common valve diseases and frequently coexist^[1-3]^. Multivalvular disease (MVD) is strongly associated with age, and most of these patients are not suitable candidates for simultaneous surgical treatment due to the high or prohibitive surgical risk. The transcatheter aortic valve implantation (TAVI) has emerged as a safe minimally invasive treatment for aortic stenosis and became a treatment of choice in patients deemed high risk for surgical aortic valve replacement (SAVR). Every fourth patient who undergoes TAVI has at least moderate MR, and improvement of MR severity has been observed in more than half of these patients^[4-8]^. The persistence of significant MR following TAVI is associated with increased morbidity and mortality^[9-11]^. However, the prognostic impact of MR after valve-in-valve TAVI (VIV-TAVI) in patients with failed bioprostheses remains unclear. The goal of our investigation was to assess the prevalence, impact of early outcomes, New York Heart Association (NYHA) functional class as well as the mortality up to five years in patients with residual moderate MR following VIV-TAVI.

## METHODS

### Study Design and Population

**Fig. 1:**
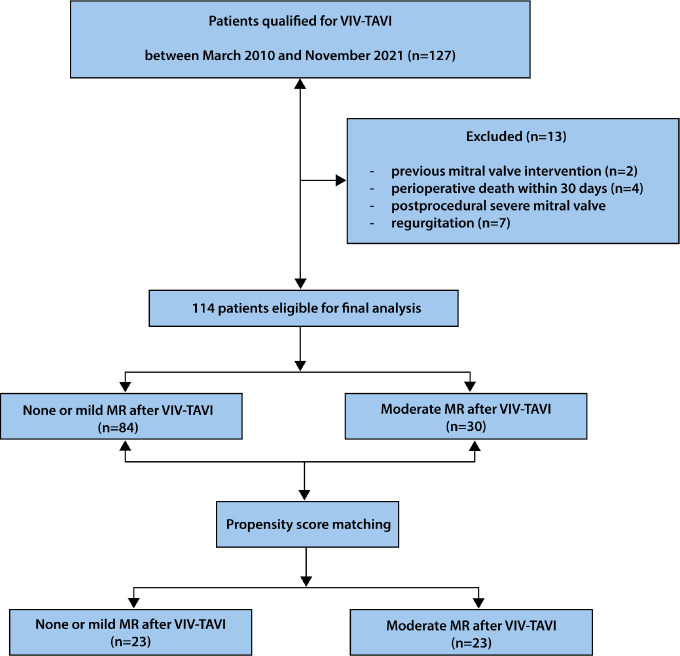
Study flow chart. Flow diagram depicting derivation of the final study population. MR=mitral regurgitation; VIV-TAVI=valve-in-valve transcatheter aortic valve implantation.

From March 2010 to November 2021, 127 patients affected by structural valve deterioration of aortic bioprostheses underwent transfemoral VIV-TAVI at Sana Heart-Center Cottbus, Germany. A total of 19 patients (15.2%) presented preoperative moderate-to-severe or severe MR, and up to 51.2% presented with at least moderate MR evidenced by echocardiography before VIV-TAVI. A comprehensive postoperative transthoracic echocardiogram after VIV-TAVI (pre-discharge) was routinely performed. Seven patients with postoperative severe MR, two patients with previous mitral valve intervention, and four patients who died before postoperative echocardiography were excluded from this study. The remaining 114 subjects were divided into two groups according to the degree of MR: those with none or mild MR after VIV-TAVI and those with moderate MR. Inclusion and exclusion criteria are presented in the flow chart (Figure 1). Pre, intra, and postoperative data were retrospectively analyzed from the hospital database and complete follow-up was performed mainly by primary care physicians with a few interviews conducted by phone, with a mean period of 4.8 years (five months - 12 years). All clinical endpoints were defined according to the current standard for definition of the events in TAVI represented by the Valve Academic Research Consortium-3 (VARC-3) criteria^[12]^. MR was defined according to the European Society of Cardiology guidelines^[8]^.

### VIV-TAVI Procedure

All individuals were considered at high operative risk or had contraindications for conventional surgical reoperation. A multidisciplinary local heart team consisting of an interventional cardiologist, clinical cardiologist, cardiac surgeon, and cardiac anesthesiologist carefully discussed the treatment strategy. All patients included for final analysis underwent VIV-TAVI with transfemoral access and self-expandable Medtronic device (Medtronic, Minneapolis, Minnesota, United States of America). Conscious sedation with local anesthesia was possible in 93% of patients. Twenty-nine initial procedures were performed with the Medtronic CoreValve™ (Medtronic, Minneapolis, Minnesota, United States of America), and the rest with CoreValve™ Evolut™ R valves (Medtronic). Most of the procedures (75.4%) were performed with an implantation level 4 mm below the neo-anulus, and the implantation depth did not differ in between the groups before and after propensity score matching (PSM). Thirty-eight patients with CoreValve™ Evolut™ R underwent repositioning of the prosthesis to optimize the position and in sixteen patients, the repositioning was performed ≥ 2 times. Predilatation was performed in all procedures, and postdilatation was required in seven patients (6.1%). Eligibility for VIV-TAVI, access route, type, and diameter of prosthesis were selected according to the routinely performed electrocardiographic gated multislice computed tomography with dedicated imaging software: either OsiriX (Pixmeo, Geneva, Switzerland) or 3mensio Valves (Pie Medical Imaging BV, Maastricht, the Netherlands).

### Statistical Analysis

Continuous variables were expressed as the means ± standard deviation (SD) (normally distributed data) or medians with interquartile range (non-normally distributed data), while categorical variables were expressed as numbers and percentages. For continuous data, Student’s *t*-test or Mann-Whitney U test were used for between-groups comparisons, while categorical variables were compared with Pearson’s χ^2^ test. To reduce the risk of selection due to the observational character of the study, a PSM was used between the groups of patients with residual moderate mitral valve regurgitation and without significant MR after VIV-TAVI. Propensity scores (PS) were generated from a logistic regression model based on the European System for Cardiac Operative Risk Evaluation II, atrial fibrillation, preoperative moderate and higher MR, preoperative higher or moderate tricuspid valve regurgitation, left atrial size, and left ventricular end-diastolic diameter and left ventricular end-systolic diameter measurements. Patients were then matched in 1:1 fashion using caliper matching method without replacement with a caliper width of 0.2 SD of the logit of the PS^[13,14]^. The balance of the covariates was tested using standardized mean difference (SMD). Statistical guidelines suggest a meaningful covariate balance of the variables used to generate the PS between the two groups to be between -0.1 < SMD < 0.1^[13]^. A survival analysis was performed according to the Kaplan-Meier method with the log-rank test used for comparison between groups. Statistical significance was assumed at *P*<0.05. Statistical analyses were conducted with the STATISTICA™ version 13 for Windows software (TIBCO StatSoft, Inc., Tulsa, Oklahoma, United States of America).

### Ethics Approval

This study was approved by the Ethics Committees of the State Chambers of Physicians in Cottbus, Germany (S34(bB)/2020).

## RESULTS

### Patients’ Characteristics

Postprocedural MR improved in 51 (42.1%) patients. There was no improvement in 70 (57.9%) patients of which six (5%) presented worse MR at discharge as compared to pre-procedure echocardiography. A total of 114 patients were included in the final analysis (44.7% female, mean age 79.7±5.6 years). Patients with moderate MR after VIV-TAVI presented a higher operative risk, underwent more often several cardiac surgeries, and they were also burdened with more comorbidities such as peripheral arterial disease. Preoperative echocardiography reveled worse left ventricular ejection fraction (LVEF), more often tricuspid valve regurgitation, increased left ventricular structural dimension, and greater left atrium in moderate MR-group. PSM yielded 23 matched pairs for final comparison (all demographics and preoperative clinical data of the matched subgroups were similar). Main clinical and preoperative echocardiographic characteristics of the global population according to the baseline degree of MR are summarized in Table 1.

**Table 1:** Patient demographic characteristics and preoperative echocardiographic findings.

Clinical characteristics^*^	Overall (n=114)	Mitral valve regurgitation after VIV-TAVI						
		Before PSM			After PSM			
		None/mild MR (n=84)	Moderate MR (n=30)	*P*-value^**^	None/mild MR (n=23)	Moderate MR (n=23)	*P*-value	SMD
Age, years	79.7±5.6	79.3±5.4	80.8±6.0	0.227	79.7±4.7	80.1±6.0	0.808	
Female	51 (44.7%)	38 (45.2%)	13 (43.4%)	0.857	6 (26.1%)	7 (30.4%)	0.743	
BMI, kg/m^2^	27.4±4.7	27.9±4.7	26.3±4.7	0.110	26.6±4.6	27.0±4.8	0.783	
EuroSCORE II, %	10.7±7.8	9.2±4.6	15.1±12.3	< 0.001	11.1±4.8	11.1±4.8	0.999	0.00
Preoperative NYHA class III/IV	97 (85.1%)	69 (82.1%)	28 (93.3%)	0.140	20 (87.0%)	21 (91.3%)	0.638	
Preoperative clinical data								
Time after index SAVR, years	9.8±4.2	9.8±4.1	10.0±4.5	0.837	10.6±4.4	9.4±3.6	0.335	
Previous PCI/CABG	59 (51.8%)	39 (46.4%)	20 (66.7%)	0.057	10 (43.5%)	16 (69.6%)	0.743	
Previous cardiac surgery > 1	8 (7.0%)	3 (3.6%)	5 (16.7%)	0.016	1 (4.3%)	3 (13.0%)	0.295	
Previous permanent pacemaker	28 (24.6%)	18 (21.4%)	10 (33.3%)	0.194	6 (26.1%)	6 (26.1%)	1.000	
Atrial fibrillation	59 (51.8%)	41 (48.8%)	18 (60.0%)	0.292	15 (65.1%)	12 (52.2%)	1.000	
Stroke	16 (14.0%)	11 (13.1%)	5 (16.7%)	0.629	4 (17.4%)	4 (17.4%)	1.000	
PAD	19 (16.7%)	10 (11.9%)	9 (30.0%)	0.022	3 (13.0%)	6 (26.1%)	0.265	
Severe pulmonary hypertension^1^	6 (5.3%)	3 (3.6%)	3 (10.0%)	0.176	2 (8.7%)	0 (0%)	0.148	
Renal impairment^2^								
Moderate	38 (33.3%)	32 (38.1%)	6 (20.0%)	0.581	9 (39.1%)	6 (26.1%)	0.312	
Severe	65 (57.0%)	44 (52.4%)	21 (70.0%)		13 (56.5%)	14 (60.9%)		
Dialysis	5 (4.4%)	3 (3.6%)	2 (6.7%)		1 (4.3%)	2 (8.7%)		
COPD	17 (14.9%)	11 (13.1%)	6 (20.0%)	0.362	1 (4.3%)	5 (21.7%)	0.080	
Insulin-dependent diabetes mellitus	16 (14.0%)	11 (13.1%)	5 (16.7%)	0.629	2 (8.7%)	4 (17.4%)	0.381	
Emergency procedure^3^	5 (4.4%)	2 (2.4%)	3 (10.0%)	0.080	2 (8.7%)	2 (8.7%)	1.000	
Preoperative echocardiographic parameters								
Mechanism of aortic bioprosthetic failure								
Predominant stenosis	81 (71.1%)	60 (71.4%)	21 (70.0%)	0.882	11 (47.8%)	17 (73.9%)	0.070	
Predominant regurgitation	33 (28.9%)	24 (28.6%)	9 (30.0%)	0.882	12 (52.2%)	6 (26.1%)	0.070	
AV mean gradient, mmHg	35.5±17.4	36.0±17.5	33.9±17.3	0.583	27.2±14.9	34.9±12.6	0.067	
AVA (cm^2^)	0.80±0.30	0.82±0.30	0.72±0.29	0.175	0.86±0.27	0.71±0.265	0.082	
LVEF, %	51.0±10.2	52.5±9.6	46.7±10.7	0.007	46.6±10.1	47.3±9.7	0.825	
TAPSE, mm	18.1±4.3	18.5±4.3	17.4±4.3	0.335	19.0±3.4	17.1±3.9	0.170	
Preoperative ≥ moderate MR	54 (47.4%)	30 (35.7%)	24 (80.0%)	< 0.001	18 (78.3%)	18 (78.3%)	1.000	0.00
Preoperative ≥ moderate TR	30 (26.3%)	17 (20.2%)	13 (43.3%)	0.014	8 (34.8%)	9 (39.1%)	0.760	-0.09
LVEDd (mm)	53.3±8.6	52.0±8.6	57.6±10.8	0.023	56.7±6.5	56.0±8.7	0.784	0.01
LVESd (mm)	38.7±9.7	36.8±9.2	43.8±13.9	0.015	42.3±8.2	42.2±10.8	0.968	0.00
LA (mm)	45.6±6.2	44.3±7.9	48.9±6.6	0.025	48.6±8.0	48.8±6.7	0.942	0.00

* Continuous variables are presented as the means ± standard deviation whereas categorical data as the numbers (n) with percentages (%);

** *P*-value < 0.05 considered as of statistical significance

1 Pulmonary artery systolic pressure > 60 mmHg

2 Moderate and severe renal impairment defined as estimated glomerular filtration rate (eGFR) > 50 < 85 ml/min/1.73m^2^ and eGFR < 50 ml/min/1.73m^2^, respectively

3 Operation before the beginning of the next working day after decision to operate

AV=aortic valve; AVA=aortic valve area; BMI=body mass index; CABG=coronary artery bypass grafting; COPD=chronic obstructive pulmonary disease; EuroSCORE=European System for Cardiac Operative Risk Evaluation; LA=left atrium; LVEDd=left ventricular end-diastolic diameter; LVEF=left ventricular ejection fraction; LVESd=left ventricular end-systolic diameter; MR=mitral regurgitation; NYHA=New York Heart Association; PAD=peripheral artery disease; PCI=percutaneous coronary intervention; PSM=propensity score matching; SAVR=surgical aortic valve replacement; SMD=standardized mean difference; TAPSE=tricuspid annular plane systolic excursion; TR=tricuspid regurgitation; VIV-TAVI=valve-in-valve transcatheter aortic valve implantation

### In-Hospital Period

Technical indices such as operative time, fluoroscopy time, contrast load, failed bioprosthesis, or TAVI valve size did differ between groups and did not impact on postoperative mitral valve insufficiency. Neither Chimney technique nor bioprosthetic aortic scallop intentional laceration (BASILICA) technique nor bioprosthetic valve fracture were performed in the analyzed population. No significant differences were found between groups before and after matching in terms of intensive care unit stay, hospital stay, transient ischemic attack, stroke, postprocedural new dialysis, new-onset atrial fibrillation, myocardial infarction, and permanent pacemaker implantation. All technical aspects and the incidences of serious adverse events according VARC-3 criteria are summarized in Table 2.

**Table 2:** Procedure-related variables, early outcomes according to VARC-3 definitions and echocardiographic findings at discharge.

Procedure-related variables	Overall (n=118)	Mitral valve regurgitation after VIV-TAVI					
		Before PSM			After PSM		
		None/mild MR (n=84)	Moderate MR (n=30)	*P*-value^**^	None/mild MR (n=23)	Moderate MR (n=23)	*P*-value
Operative time, min	51±22	51±24	50±18	0.794	57±29	50±16	0.278
Contrast load, cc	183±83	186±87	175±68	0.537	206±64	171±64	0.077
Fluoroscopy time, min	14±13	15±14	13±6	0.492	15±10	12±6	0.298
Local anesthesia	106 (93.0%)	79 (94.0%)	27 (90%)	0.456	20 (87.0%)	22 (95.7%)	0.295
VARC-3 variables^*^	Clinical events according to VARC-3 definitions						
ICU stay, days	1 (1-1)	1 (1-1)	1 (1-1)	0.151	1 (1-1)	1 (1-1)	1.000
Hospital stay, days	6 (5-7.25)	6 (5-7)	6 (5-8)	0.406	6 (5-7)	6 (5.5-7.5)	0.887
Myocardial infarction	1 (0.9%)	1 (1.2%)	0 (0%)	0.548	0 (0%)	0 (0%)	1.000
Cardiac tamponade	1 (0.9%)	1 (1.2%)	0 (0%)	0.548	0 (0%)	0 (0%)	1.000
TIA	1 (0.9%)	1 (1.2%)	0 (0%)	0.548	0 (0%)	0 (0%)	1.000
Stroke	5 (4.4%)	5 (6.0%)	0 (0%)	0.172	1 (4.3%)	0 (0%)	0.312
AKI	4 (3.5%)	2 (2.4%)	2 (6.7%)	0.273	1 (4.3%)	0 (0%)	0.312
Permanent pacemaker implantation	4 (3.5%)	2 (2.4%)	2 (6.7%)	0.274	0(0%)	2 (8.7%)	0.148
New-onset atrial fibrillation	2 (1.8%)	1 (1.2%)	1 (3.3%)	0.443	1 (4.3%)	1 (4.3%)	1.000
Death occurring > 30 days but < 1 year after the index hospitalization	11 (9.3%)	3 (3.6%)	4 (13.3%)	0.055	0 (0%)	3 (13.0%)	0.073
Echocardiographic variables	Echocardiographic parameters at discharge^***^						
Paravalvular regurgitation							
Mild	39 (34.2%)	27 (32.1%)	12 (40.0%)	0.436	11 (47.8%)	9 (39.1%)	0.552
Moderate	5 (4.4%)	3 (3.6%)	2 (6.7%)	0.477	2 (8.7%)	1 (4.3%)	0.550
Severe	0 (0%)	0 (0%)	0 (0%)	1.000	0 (0%)	0 (0%)	1.000
AV mean gradient, mmHg	15.1±8.4	15.7±8.1	13.4±8.9	0.199	13.5±6.5	14.4±9.8	0.710
AV peak gradient, mmHg	26.8±13.4	27.8±13.7	24.0±12.3	0.186	24.5±10.6	25.8±13.0	0.711
TR ≥ 2^o^	22 (19.3%)	16 (19.0%)	6 (20.0%)	0.910	3 (13.0%)	6 (26.1%)	0.265
LVEF, %	52.0±9.8	52.9±9.6	49.4±10.2	0.091	47.3±10.2	49.6±9.7	0.445
TAPSE, mm	17.5±4.9	17.4±5.5	17.7±3.4	0.805	16.2±4.3	17.3±3.3	0.448

* Continuous variables are presented as the means ± standard deviation or the medians with interquartile range whereas categorical data as the numbers (n) with percentages (%)

** *P*-value < 0.05 considered as of statistical significance

*** The echocardiographic variables were summarized only if the echocardiographic data of those patients were available after procedure (n=245, 97.2%), seven patients (2.8%) died before their scheduled postoperative echocardiographic examination

AKI=acute kidney injury; AV=aortic valve; ICU=intensive care unit; LVEF=left ventricular ejection fraction; MR=mitral regurgitation; PSM=propensity score matching; TAPSE=tricuspid annular plane systolic excursion; TIA=transient ischemic attack; TR=tricuspid regurgitation; VARC-3=Valve Academic Research Consortium-3; VIV-TAVI=valve-in-valve transcatheter aortic valve implantation

### Overall Hemodynamic Results

At discharge, the mean postoperative transvalvular pressure gradient was 15.1±8.4 mmHg and was comparable between both groups. Paravalvular leak (PVL) occurred in 44 (38.6%) patients, however, nearly 90% of them presented mild PVL, and we did not observe any severe PVL after VIV-TAVI. Both LVEF and tricuspid annular plane systolic excursion did not significantly change after VIV-TAVI, and there were no differences between groups before and after PSM (Table 2).

### Long-Term Mortality

**Fig. 2:**
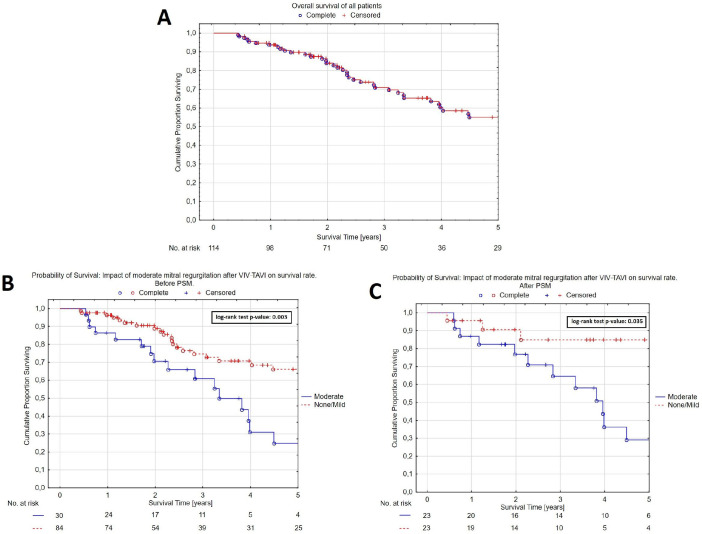
A) Kaplan-Meier survival curve of all patients. B) None/mild vs. moderate mitral regurgitation following valve-in-valve transcatheter aortic valve implantation (VIV-TAVI) before propensity score matching (PSM). C) None/mild vs. moderate mitral regurgitation following VIV-TAVI after PSM.

In the follow-up period, 71 (62.3%) patients survived, 59 (70.2%) of them in the none/mild MR group, and 12 (40.0%) in the moderate MR group. The overall survival probabilities at one, three, and five years were 93.7%, 71.0%, and 53.8% (Figure 2A). In the unmatched cohort, survival probabilities at one, three, and five years were 96.3% *vs.* 86.3%, 74.6% *vs.* 60.9%, and 66.0% *vs.* 25.0 % in the none to mild MR group *vs.* moderate MR group, respectively (log-rank *P*=0.003) (Figure 2B). In the matched cohort, survival probabilities at one, three, and five years were 95.7% *vs.* 87.0%, 85.0% *vs.* 64.5%, and 85.0% *vs.* 29.0% in the none to mild MR group *vs.* moderate MR group, respectively (log-rank *P*=0.035) (Figure 2C). None of the patients required repeated TAVI or MitraClip™ device implantation following VIV-TAVI during the follow-up period.

### NYHA Functional Class at Follow-up Period

NYHA class I or II was observed in 60 out of 71 (84.5%) survivors. Among survivors, we noted increased incidence of heart failure categorized as NYHA class III in the moderate MR group (n=5/12 [41.7%] *vs.* 6/59 [10.2%], *P*=0.006).

## DISCUSSION

Over the past few years, we observed a trend towards increased numbers of implantation of bioprosthetic aortic valves, which in turn led to an increase in the number of patients with degenerated bioprostheses, especially in younger patients^[15,16]^. Those patients may require redo-SAVR or VIV-TAVI. Around 60% of the patients who underwent VIV-TAVI in our study presented at least moderate MR at baseline. The present study showed that residual moderate MR after VIV-TAVI is not associated with worse early outcomes, however, it was associated with increased mortality at five years and worse NYHA class in survivors.

Several researchers analyzed the impact of baseline MR and MR-severity improvement on outcomes after aortic valve intervention, however, the results remain controversial. In 2011, Harling et al.^[17]^ published a meta-analysis identifying 17 studies (3,053 patients) and assessed the influence of co-existing MR on outcomes after SAVR. An improvement of MR following SAVR was observed in 55.5%. None-mild MR was associated with higher 30-day, three-, five-, and 10-year survival following SAVR when compared to patients with moderate-severe MR (*P*=0.002, *P*<0.0001, *P*<0.00001, and *P*=0.02, respectively). The authors emphasized the need of further randomized trials to assess the effect of mitral intervention *vs.* non-intervention in patients with concomitant baseline moderate MR. Barreiro et al.^[18]^ conducted a retrospective review of 408 consecutive patients who underwent isolated SAVR and recorded moderate MR in only 17.2% of patients. Preoperative moderate MR was recognized as an independent risk factor for mortality (*P*=0.032) and functional outcome in elderly patients (≥ 70 years old). The authors observed a higher survival rate at five years in patients with improved MR after the surgery (72% *vs.* 58%), however, the difference was statistically not significant due to the limited postoperative data. The role of untreated mild-moderate MR was evaluated by Takeda et al.^[19]^ who conducted a retrospective study of 193 patients who underwent isolated SAVR between 1993 and 2007. They did not find any significant differences in mortality regardless baseline MR grade (*P*=0.49). However, patients with mild-moderate MR presented lower freedom from readmission for heart failure at 10 years in comparison with patients with non-trivial MR at baseline (23% *vs.* 83%; *P*=0.002). Multivariate analysis identified mild-moderate MR at baseline as independent predictors of heart failure (*P*=0.012). In 2013, Barbani et al.^[20]^ analyzed data from the Placement of AoRtic TraNscathetER Valves (PARTNER) Registry to assess the impact of preoperative MR on outcomes after SAVR (n=299) and TAVI (n=331). They observed higher two-year mortality rates in patients with preoperative moderate MR before SAVR (49.1% *vs.* 27.9%, *P*<0.01), however, such deleterious effect on mortality was not observed in the TAVI population (37.0% *vs.* 32.7%, *P*=0.58). Opposite results were presented by Bedogni et al.^[21]^ and Gianini et al.^[22]^ who revealed association of higher mortality after self-expandable TAVI valves in patients with baseline MR greater than mild. Malaisrie et al.^[23]^ focused on intermediate risk patients from the PARTNER Registry (n=2,032) with preoperative significant MR in patients who underwent SAVR or TAVR. They observed improvement of MR severity in 47% of patients. The authors demonstrated better 30-day survival rate in patients with ≤ mild MR after SAVR (8.0% *vs.* 3.5%, *P*=0.01), however, this difference was not seen in the TAVI population (2.7% *vs.* 3.1%, *P*=0.78). In both SAVR and TAVI procedures, baseline ≥ moderate MR yielded worse two-year outcomes.

Joo et al.^[24]^ focused on persistency of MR after SAVR and observed worse 10-year survival after SAVR (93.1% *vs.* 77.8 %, respectively, *P*=0.036) in patients with residual MR. They suggested that postoperative residual MR could be more important than preoperative MR in the prediction of long-term results in functional MR after isolated SAVR. In the meta-analysis of eight studies performed by Chakravarty et al.^[10]^, involving 8,927 patients, 22% of patients before TAVI presented moderate-severe MR, and the MR improvement rate after the procedure amounted to 61%. They were the first who observed increased one-year mortality in patients with residual moderate-severe MR, compared to residual none-mild MR after TAVI (risk ratio 1.48, 95% confidence interval [CI] 1.31 to 1.68, *P*<0.00001). The authors emphasized the importance of residual MR after TAVI, and the results suggested that the treatment of persistence MR following TAVI could potentially optimize outcomes. The importance of postoperative MR has been also reported by Mauri et al.^[11]^. They confirmed that patients with severe MR at baseline had poorer survival, and even improvement to moderate MR after TAVI was associated with increased risk for death despite the improvement. Mauri et al.^[11]^ concluded that the degree of MR after TAVI has a crucial influence on long-term results and not the improvement itself.

The combination of failed aortic bioprostheses in conjunction with MR was analyzed in a few studies. Hahn et at.^[25]^ examined a multicenter population from the PARTNER-2 Aortic Valve-in-Valve registry and reported a decrease in the numbers of moderate or severe MR after the procedure (from 34.7% at baseline to 15.3% early after the procedure and to 4% at five years after VIV-TAVI). Murdoch et al.^[26]^ evaluated 339 patients from the same registry and assessed the impact of baseline moderate MR on outcomes after VIV-TAVI. They recorded moderate MR before VIV-TAVI in 32.7% of patients, and the authors did not find baseline moderate MR as a predictor of long-term adverse outcomes in the VIV-TAVI population (one-year mortality ≤ mild MR *vs.* moderate MR: 15.5% *vs.* 15.3%, *P*=0.98; and two-year mortality: 26.5% *vs.* 23.5%, *P*=0.67). The other large analysis was conducted by Tuzcu et al.^[27]^ who compared data of 1,150 patients after VIV-TAVI with 2,259 patients after native TAVI from The Transcatheter Valve Therapy (TVT) Registry. The authors observed a significantly higher rate of severe MR before VIV-TAVI compared to native TAVI (39.3% *vs.* 30.6%, *P*<0.001). Tuzcu et al.^[27]^ found that preoperative MR (≥ moderate) was not associated with one-year mortality after VIV-TAVI (hazard ratio 0.97, 95% CI 0.62-1.51, *P*=0.88).

Data of both large registries which analyzed failed bioprostheses (PARTNER-2 Aortic Valve-in-Valve registry and TVT registry) observed no impact of preoperative MR on one-year mortality. It is worth mentioning that both studies did not consider the impact of postoperative MR on outcomes. In the current study, the presence of residual moderate MR after VIV-TAVI was not associated with worse early results, however, our investigation found a negative impact of MR on long-term survival and worse functional status in patients with residual moderate MR following VIV-TAVI.

The majority of patients who undergo aortic valve reintervention have MVD and they are mostly non suitable candidates for double-valve open-heart surgery. Transcatheter mitral valve repair procedure following VIV-TAVI might be a solution to optimize outcomes in these high-risk patients, however, this assumption goes beyond what our present data allow us to affirm and further large randomized trials are warranted.

### Limitations

The main limitation of this study relates to the fact that it is a retrospective non-randomized single-center study with a relatively limited sample size. The mechanism of MR is not considered in this study, although the current investigation analyzed heterogeneous group of patients with 19 different valve types used for the primary aortic valve replacement and various combinations of types and sizes of TAVI valves. During the study period, which lasted over 10 years, we observed many technical and procedural improvements in TAVI procedures, that may affect the results of the study. A further point is the learning curve, which may influence the worse outcome in the earlier years of the study period. Moreover, there were no monitoring board or core laboratory available for echocardiographic analysis, that were consequences of a retrospective nature of our study. Future multicenter randomized studies with larger samples and echocardiographic follow-up periods are warranted.

## CONCLUSION

Mitral valve regurgitation is common in patients with degenerated aortic bioprostheses and improvement of MR is observed in over 40% of patients following VIV-TAVI. Residual moderate MR after VIV-TAVI is not associated with worse early outcomes, however, it was associated with increased mortality at five years of follow-up and worse NYHA class among survivors. Therefore, further randomized large studies are necessary to confirm the association of residual MR following VIV-TAVI with adverse outcomes and to plan possible intervention in order to reduce its impact.

## Abbreviations

AKI: = Acute kidney injury
AV: = Aortic valve
AVA: = Aortic valve area
BMI: = Body mass index
CABG: = Coronary artery bypass grafting
CI: = Confidence interval
COPD: = Chronic obstructive pulmonary disease
eGFR: = Estimated glomerular filtration rate
EuroSCORE: = European System for Cardiac Operative Risk Evaluation
ICU: = Intensive care unit
LA: = Left atrium
LVEDd: = Left ventricular end-diastolic diameter
LVEF: = Left ventricular ejection fraction
LVESd: = Left ventricular end-systolic diameter
MR: = Mitral regurgitation
MVD: = Multivalvular disease
NYHA: = New York Heart Association
PAD: = Peripheral artery disease
PARTNER: = Placement of AoRtic TraNscathetER Valves
PCI: = Percutaneous coronary intervention
PS: = Propensity score
PSM: = Propensity score matching
PVL: = Paravalvular leak
SAVR: = Surgical aortic valve replacement
SD: = Standard deviation
SMD: = Standardized mean difference
TAPSE: = Tricuspid annular plane systolic excursion
TAVI: = Transcatheter aortic valve implantation
TIA: = Transient ischemic attack
TR: = Tricuspid regurgitation
TVT: = Transcatheter Valve Therapy
VARC-3: = Valve Academic Research Consortium-3
VIV-TAVI: = Valve-in-valve transcatheter aortic valve implantation
